# Surgical Outcomes, Health Care Utilization, and Costs Associated with Staple Line Buttressing Among Primary Sleeve Gastrectomy Patients

**DOI:** 10.1007/s11695-020-04917-2

**Published:** 2020-09-10

**Authors:** Sanjoy Roy, Yuexi Wang, Rajesh Mallampati, Stephen Johnston

**Affiliations:** 1 Johnson & Johnson, Inc., New Brunswick, NJ USA; 2 STATinMED Research, Ann Arbor, MI USA

**Keywords:** Staple line buttress, Laparoscopic sleeve gastrectomy, Surgical outcome, Health care costs

## Abstract

**Purpose:**

Staple line buttressing is a method of reinforcing surgical staple lines using buttress materials. This study evaluated surgical outcomes, hospital utilization, and hospital costs associated with staple line buttressing among patients who underwent primary laparoscopic sleeve gastrectomy (PLSG) in the United States.

**Methods:**

This was a retrospective cohort study using Premier Healthcare Database data from January 1, 2012 to December 31, 2017. Patients aged ≥ 18 years who underwent PLSG were selected and assigned to buttress or non-buttress cohorts based on the use of buttress material during their hospitalization for PLSG (index). Propensity score matching (PSM) was conducted to balance patient demographic and clinical characteristics between the cohorts. Generalized estimating equation models were used to compare the clinical and economic outcomes of the matched buttress and non-buttress users during the index hospitalization.

**Results:**

A total of 38,231 buttress and 27,349 non-buttress patients were included in the study. After PSM, 24,049 patients were retained in each cohort. Compared with non-buttress cohort, the buttress cohort patients had a similar rate of in-hospital leaks (0.28% vs 0.39%; *p* = 0.160) and a lower rate of bleeding (1.37% vs 1.80%, *p* = 0.015), transfusion (0.56% vs 0.77%, *p* = 0.050), and composite bleeding/transfusion (1.57% vs 2.04%, *p* = 0.019). Total costs ($12,201 vs $10,986, *p* < 0.001) and supply costs ($5366 vs $4320, *p* < 0.001) were higher in the buttress cohort compared with the non-buttress cohort.

**Conclusions:**

Staple line buttressing was associated with an improvement in complication rates for bleeding and transfusion. Total and supply costs were higher in the buttress cohort, necessitating further research into cost-effective buttressing materials.

**Electronic supplementary material:**

The online version of this article (10.1007/s11695-020-04917-2) contains supplementary material, which is available to authorized users.

## Introduction

Obesity is an increasingly prevalent problem in the United States [1]. Laparoscopic sleeve gastrectomy (LSG) is widely recognized as a primary bariatric procedure for treating patients who are obese, with or without select medical conditions [2, 3]. LSG is proven to be an effective procedure with many advantages; however, complications such as bleeding, staple line leaks, and abscess formation may occur [4, 5].

Staple line buttressing is a method of reinforcing surgical staple lines using buttress materials in bariatric surgical procedures [6, 7]. Buttress materials are broadly classified as absorbable, semi-absorbable, and non-absorbable [8]. Absorbable buttresses are especially advantageous as they do not leave a permanent foreign object in a patient’s body, potentially reducing post-surgical complications [8].

Buttressing the staple line of LSGs is known to provide favorable outcomes in terms of surgical complications and health care utilization. Evidence from existing studies shows a reduction in bleeding, overall complications, and other adverse events associated with the use of buttressing in LSG [6, 7, 9–11]. One study that examined patients undergoing LSG in the Metabolic and Bariatric Surgery Accreditation and Quality Improvement Program found that patients with buttressing had a 30% lower rate of postoperative bleeding [10]. In another meta-analysis, there was a non-statistically significant reduction in postoperative leaks associated with buttress use among patients who underwent LSG [11]. Buttress use has also been associated with shorter operative times, shorter hospitalization length of stay (LOS), and fewer morbidities [6, 12]. These advantages could outweigh any additional costs incurred from buttress use.

While study outcomes associated with staple line buttressing have been evaluated in randomized clinical trials (RCTs) and prospective cohort studies with small sample sizes, there is a lack of recent real-world evidence among patients undergoing LSG in the United States. Utilizing a large nationwide healthcare database, this study sought to examine the associations of buttress use with surgical outcomes, hospital utilization, and hospital costs among patients who underwent a primary LSG.

## Methods

### Data Source

This was a retrospective cohort study using Premier Healthcare Database (PHD) data from January 1, 2012 through December 31, 2017 [13]. PHD is a large, service-level, all-payer US hospital database that contains information on inpatient discharges, primarily from geographically diverse non-profit, non-governmental, and community/teaching hospitals and health systems from rural and urban areas. It contains > 970 contributing hospitals located throughout the country and includes information on hospital and visit characteristics as well as patient data from standard hospital discharge billing files. The database contains data from > 208 million unique patients and > 2.5 million daily service records for an average of > 5 million deidentified hospital discharges per year. Multiple health care encounters can be tracked within a hospital for any patient. The database contains deidentified patient information, is Health Insurance Portability and Accountability Act (HIPAA) compliant, and is considered exempt from institutional review board (IRB) approval [14]. The database has been used previously to evaluate surgical outcomes [15, 16]. A commercially available data license is required for access to the database and may be obtained by contacting the Premier Healthcare Database directly.

### Patient Selection

Patients with evidence of hospital discharge records including International Classification of Diseases, Ninth Revision, Clinical Modification (ICD-9-CM) and ICD Tenth Revision, Procedure Coding System (ICD-10-PCS) procedure codes for LSG surgery (ICD-9-CM: 43.82; ICD-10-PCS: 0DB64Z3) during the study identification period (April 1, 2012 to September 30, 2017) were selected. The first observed inpatient visit with evidence of the procedure was designated as the index hospitalization. Patients were required to be aged ≥ 18 years on the year of index hospitalization. Patients with LSG as a secondary procedure and those with LSG any time prior to their index hospitalization were excluded. Patients were also excluded if they had a non-elective admission for the index hospitalization or did not have obesity as their primary diagnosis. Finally, patients whose procedure was converted to an open procedure during the index hospitalization and those with band removal/revisions during the baseline period or index hospitalizations were excluded as well.

The final study sample was assigned to two cohorts based on evidence of buttress use ascertained from hospital chargemaster records. Patients with evidence of buttress use (both absorbable and non-absorbable) during the index hospitalization were assigned to the buttress cohort; all patients without evidence of buttress use, including those who could have undergone over-sewing during the index hospitalization, were assigned to the non-buttress cohort. All analyses were exclusively restricted to patients undergoing LSG as a primary procedure (primary, secondary, and revisional procedures excluded) conducted in hospitals for which there was at least one record of buttress use during the same year to avoid misclassification from hospitals that may use buttressing but fail to record it in their chargemaster records.

### Baseline Characteristics

Patient demographics including age, sex, race, geographic region, marital status, and payer type during the index hospitalization were examined. Each patient’s body mass index (BMI) and Charlson comorbidity index (CCI) score during the baseline period (during index hospitalization and the 3 months prior) and their year of surgery were recorded. Patient hospital characteristics included for analysis were procedure volume, hospital type (teaching/non-teaching), hospital location (urban/rural), and bed size (< 200, 200–499, or ≥ 500 beds). Procedure volume was defined as the hospital’s frequency of LSG procedures during the year of index hospitalization.

### Outcome Measures

Surgical outcomes, healthcare utilization, and costs during the index hospitalization were measured and compared between buttress and non-buttress cohorts. Surgical outcomes included in-hospital leaks, bleeding, and transfusion, identified by ICD-9/10-CM/PCS codes. A composite bleeding/transfusion outcome representing bleeding and/or transfusion events was also included. Healthcare utilization included LOS (in days) and time in the operating room (OR, in hours). Total hospital costs and those specifically related to the OR, room and board, and supplies during the index hospitalization were calculated and adjusted to 2017 US dollars; costs were measured from the hospital perspective as opposed to the payer perspective.

### Statistical Methods

#### Descriptive Analysis

Descriptive statistics were used to compare means and proportions between the buttress and non-buttress cohorts for baseline characteristics and outcome measures before propensity score matching (PSM). Standardized mean differences (SMDs) between these cohorts were calculated as 100 times the absolute value of the SMD; as per standard practice, an SMD > 10 was considered to be indicative of imbalance between cohorts [17].

PSM was used to perform a 1:1 match between study cohorts with respect to all hospital and patient characteristics described above, with a maximum caliper width of 0.01 for the absolute probability using the nearest neighbor technique without replacement. Generalized estimating equation (GEE) models, accounting for hospital-level clustering of the study outcomes, were used to compare surgical outcomes and economic outcomes between the matched buttress and non-buttress cohorts. Surgical outcomes were modeled using a binomial distribution with a logit link; LOS and OR time were modeled using negative binomial distribution, and cost outcomes were modeled using a gamma distribution with log link. Marginal effects were computed using the recycled prediction method [18]. A *p* value of < 0.05 was set as the threshold for statistical significance. All statistical analyses were conducted using the Statistical Analysis System (SAS) v.9.4. (Cary, North Carolina, USA).

## Results

### Patient and Hospital Characteristics

**Fig. 1:**
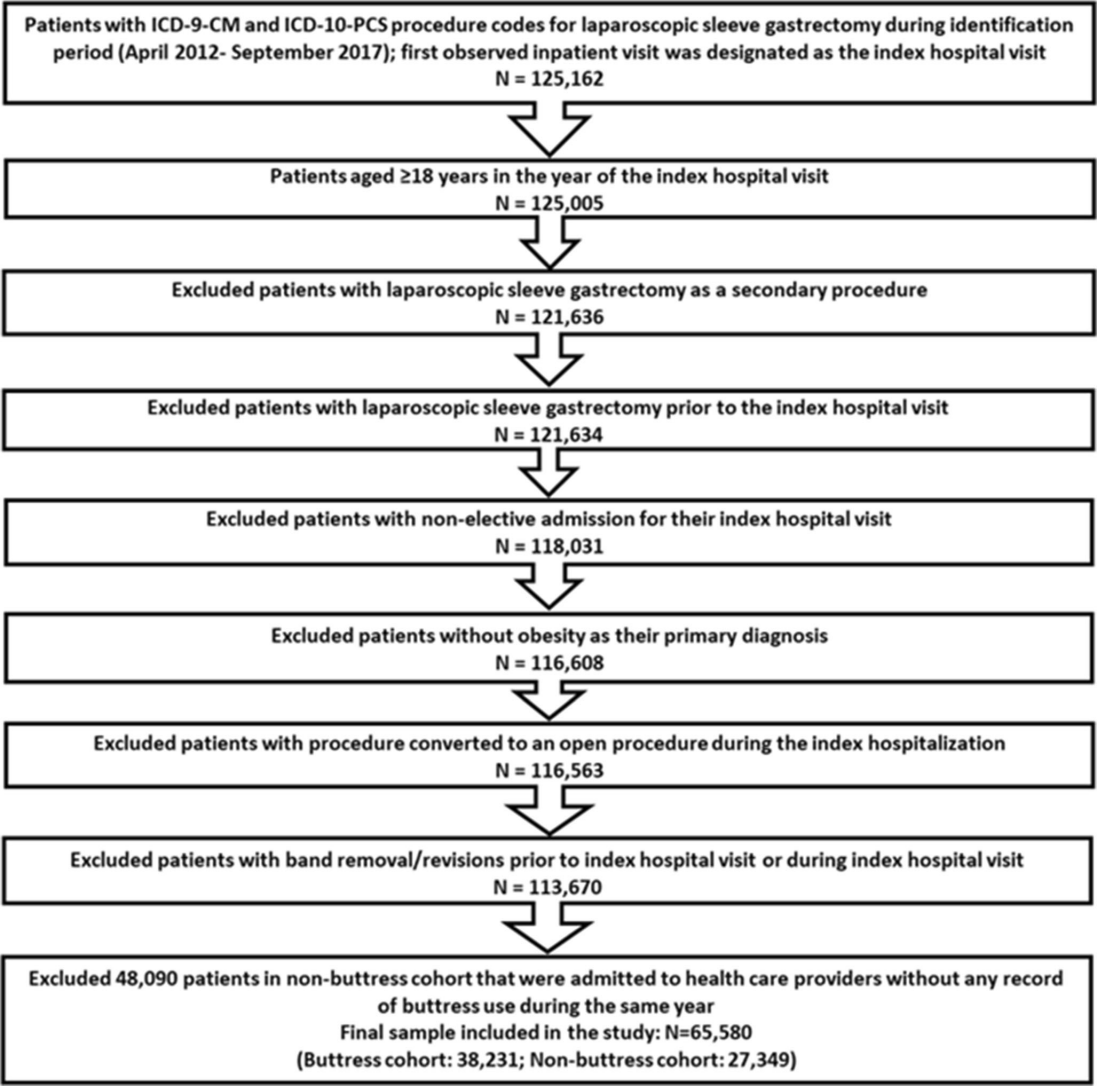
Patient selection criteria. ICD-9-CM: international classification of diseases, ninth revision, clinical modification; ICD-10-PCS: international classification of disease, tenth revision, procedure coding system

After applying selection criteria, 38,231 patients with buttress use and 27,349 patients without buttress use were included in the study (Fig. 1).

Before matching, patients in the buttress cohort had similar age (45 vs 44 years), sex (male: 20.8% vs 21.3%), marital status (married: 53.5% vs 51.2%), and geographic region compared with non-buttress cohort patients. Managed care and commercial plans (64.6% vs 65.2%) accounted for the highest proportion of payers followed by governmental plans (27.6% vs 27.8%). The proportion of Caucasian patients was slightly higher in the buttress cohort (70.7% vs 65.4%; SMD = 11.2), while the proportion of African American patients was slightly higher in the non-buttress cohort (20.9% vs 16.5%; SMD = 11.2) (Table 1).

**Table 1:** Demographic characteristics before propensity score matching

Baseline variables	Non-buttress cohort		Buttress cohort		SMD
	(*N* = 27,349)		(*N* = 38,231)		
	*N*/mean	%/SD	*N*/mean	%/SD	
Age	44	11.8	44.6	11.9	5
18–34	6329	23.1%	8296	21.7%	3.5
35–54	15,490	56.6%	21,393	56.0%	1.4
55–64	4069	14.9%	6395	16.7%	5.1
65+	1461	5.3%	2147	5.6%	1.2
Race					
African American	5711	20.9%	6318	16.5%	*11.2*
Caucasian	17,893	65.4%	27,011	70.7%	*11.2*
Other race	3389	12.4%	4667	12.2%	0.6
Unknown race	356	1.3%	235	0.6%	7.1
Sex					
Male	5820	21.3%	7955	20.8%	1.2
Female	21,529	78.7%	30,276	79.2%	1.2
Marital status					
Married	13,996	51.2%	20,441	53.5%	4.6
Single	11,234	41.1%	15,193	39.7%	2.7
Other	2119	7.7%	2597	6.8%	3.7
US geographic region					
Northeast	5415	19.8%	8200	21.4%	4.1
Midwest	5691	20.8%	7252	19.0%	4.6
South	13,319	48.7%	17,826	46.6%	4.2
West	2924	10.7%	4953	13.0%	7
Payer type					
Managed care and commercial	17,842	65.2%	24,716	64.6%	1.2
Government (Medicare, Medicaid) and other government payers	7607	27.8%	10,540	27.6%	0.5
Other (i.e., self-pay, workers’ compensation, direct employer contract, other)	1877	6.9%	2968	7.8%	3.5
Indigent and charity	23	0.1%	7	0.0%	2.9

*SD* standard deviation, *SMD* standardized mean difference

Italics = Significant difference between the study cohorts if SMD > 10

Similar CCI scores (0.64 vs 0.68) and BMI were observed between the cohorts (Table 2). Patients in the buttress cohort were more likely to be treated in a hospital with lower procedure volume, located in an urban area (93.4% vs 90.0%; SMD = 12.2) and less likely to be treated in a hospital with < 200 beds (18.8% vs 24.6%; SMD = 13.9) (Table 2).

**Table 2:** Hospital and patient clinical characteristics before propensity score matching

Baseline variables	Non-buttress cohort		Buttress cohort		SMD
	(*N* = 27,349)		(*N* = 38,231)		
	*N*/Mean	%/SD	*N*/Mean	%/SD	
Patient clinical characteristics					
Body mass index (BMI)					
<40	4982	18.2%	7105	18.6%	0.9
40–45	8727	31.9%	11,966	31.3%	1.3
45–50	5868	21.5%	8172	21.4%	0.2
50–60	5172	18.9%	7551	19.8%	2.1
60–70	1382	5.1%	1869	4.9%	0.8
≥ 70	400	1.5%	587	1.5%	0.6
Missing BMI information	818	3.0%	981	2.6%	2.6
Year of surgery					
2012	2250	8.2%	2838	7.4%	3
2013	3810	13.9%	5856	15.3%	3.9
2014	5574	20.4%	7153	18.7%	4.2
2015	6283	23.0%	8107	21.2%	4.3
2016	5927	21.7%	8552	22.4%	1.7
2017	3505	12.8%	5725	15.0%	6.2
Deyo-Charlson comorbidity index score	0.64	0.95	0.68	1.00	4.4
0 (Reference)	15,216	55.6%	20,810	54.4%	2.4
1	8789	32.1%	12,179	31.9%	0.6
2–3	2846	10.4%	4380	11.5%	3.4
4+	498	1.8%	862	2.3%	3.1
Hospital characteristics					
Procedure volume (no. procedures/year)	840.3	448.1	743.3	383.2	*23.3*
Hospital type					
Teaching	12,061	44.1%	17,510	45.8%	3.4
Non-teaching	15,288	55.9%	20,721	54.2%	3.4
Hospital location					
Urban	24,627	90.0%	35,707	93.4%	*12.2*
Rural	2722	10.0%	2524	6.6%	*12.2*
Hospital size					
< 200 beds	6715	24.6%	7206	18.8%	*13.9*
200–499 beds	13,574	49.6%	20,489	53.6%	7.9
≥ 500 beds	7060	25.8%	10,536	27.6%	3.9

*SD* standard deviation, *SMD* standardized mean difference

Italics = Significant difference between the study cohorts if SMD > 10

### Surgical Outcomes

Before PSM, there was little variation between cohorts in the proportions of in-hospital leaks (0.4% vs 0.3%; SMD = 2.4), bleeding (1.3% vs 2.1%; SMD = 6.5), transfusion (0.6% vs 0.8%; SMD = 2.5), and bleeding/transfusion (1.5% vs 2.4%; SMD = 6.5; Table 3).

**Table 3:** Descriptive outcome characteristics at index hospitalization before propensity score matching

Outcomes	Non-buttress (*N* = 27,349)		Buttress (*N* = 38,231)		SMD
Surgical outcomes^a^	*N*/Mean	%/SD	*N*/Mean	%/SD	
In-hospital leak	70	0.3%	149	0.4%	2.4
Bleeding/transfusion	635	2.4%	554	1.5%	6.5
Bleeding	567	2.1%	478	1.3%	6.5
Transfusion	211	0.8%	218	0.6%	2.5
Hospitalization utilization					
Index hospitalization length of stay, days	1.70	3.67	1.75	0.97	1.7
Operation room time during index hospitalization, hours between 30 min and 24 h (*N* = 23,512 vs. 32,060)	3.09	3.26	2.85	2.01	8.7
Hospitalization costs					
Total costs	$10,712	$5406	$11,477	$4844	14.9
Operation room	$4040	$2431	$3752	$2148	12.5
Room and board	$993	$847	$1085	$728	11.6
Supply	$4134	$3696	$4890	$3072	22.3

*SD* standard deviation, *SMD* standardized mean difference

^a^Patients with events that presented on admission were excluded from the numerator and denominator

**Fig. 2:**
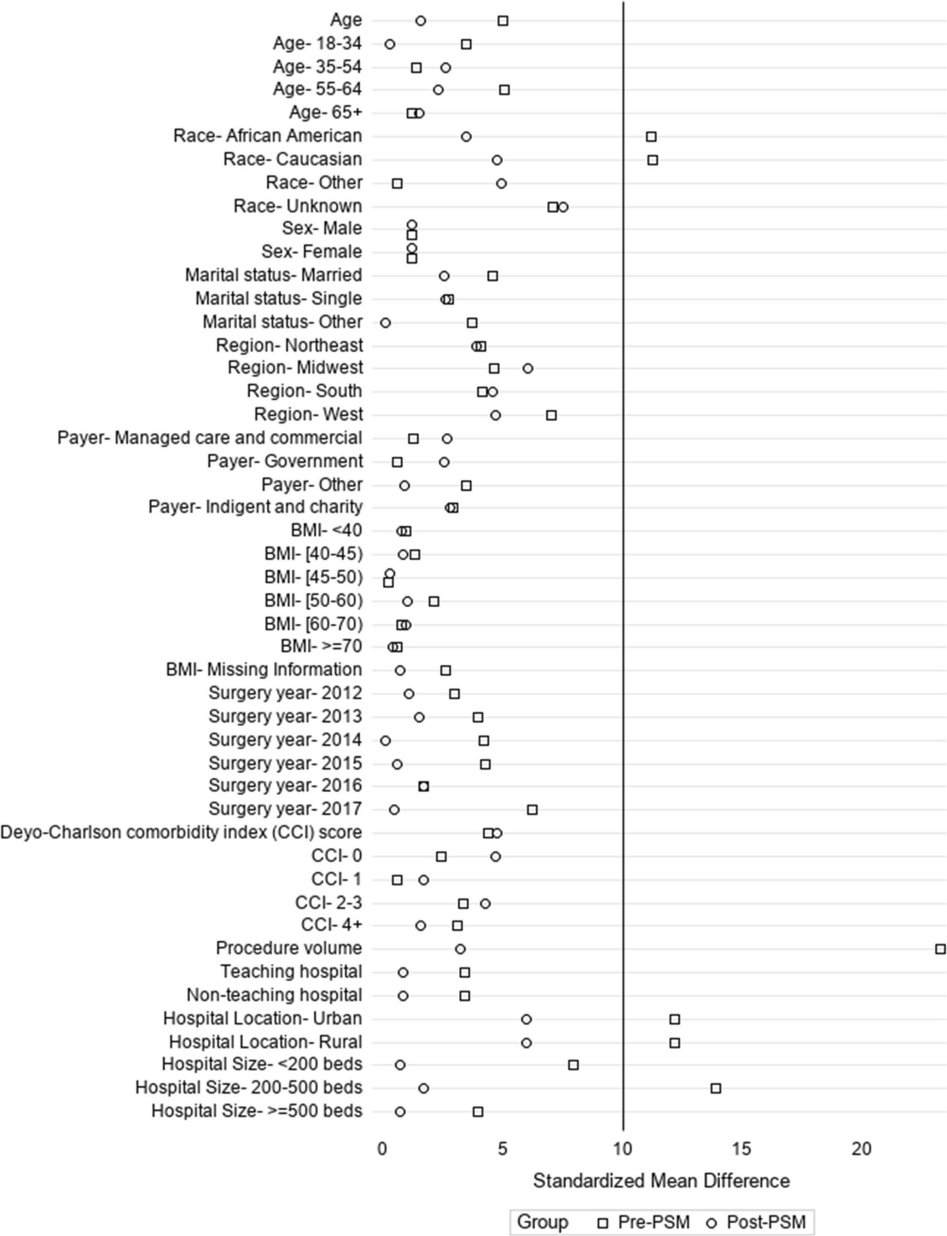
Standardized mean differences before and after propensity score matching. Mean standardized difference ≥ 10 is considered significant; BMI: body mass index; CCI: Charlson comorbidity index; PSM: propensity score matching

After PSM, there were 24,049 patients in both the buttress and non-buttress cohorts. All patient and hospital characteristics were well balanced after PSM (Fig. 2). Patient characteristics after PSM are presented in Supplemental Table 1.

**Fig. 3:**
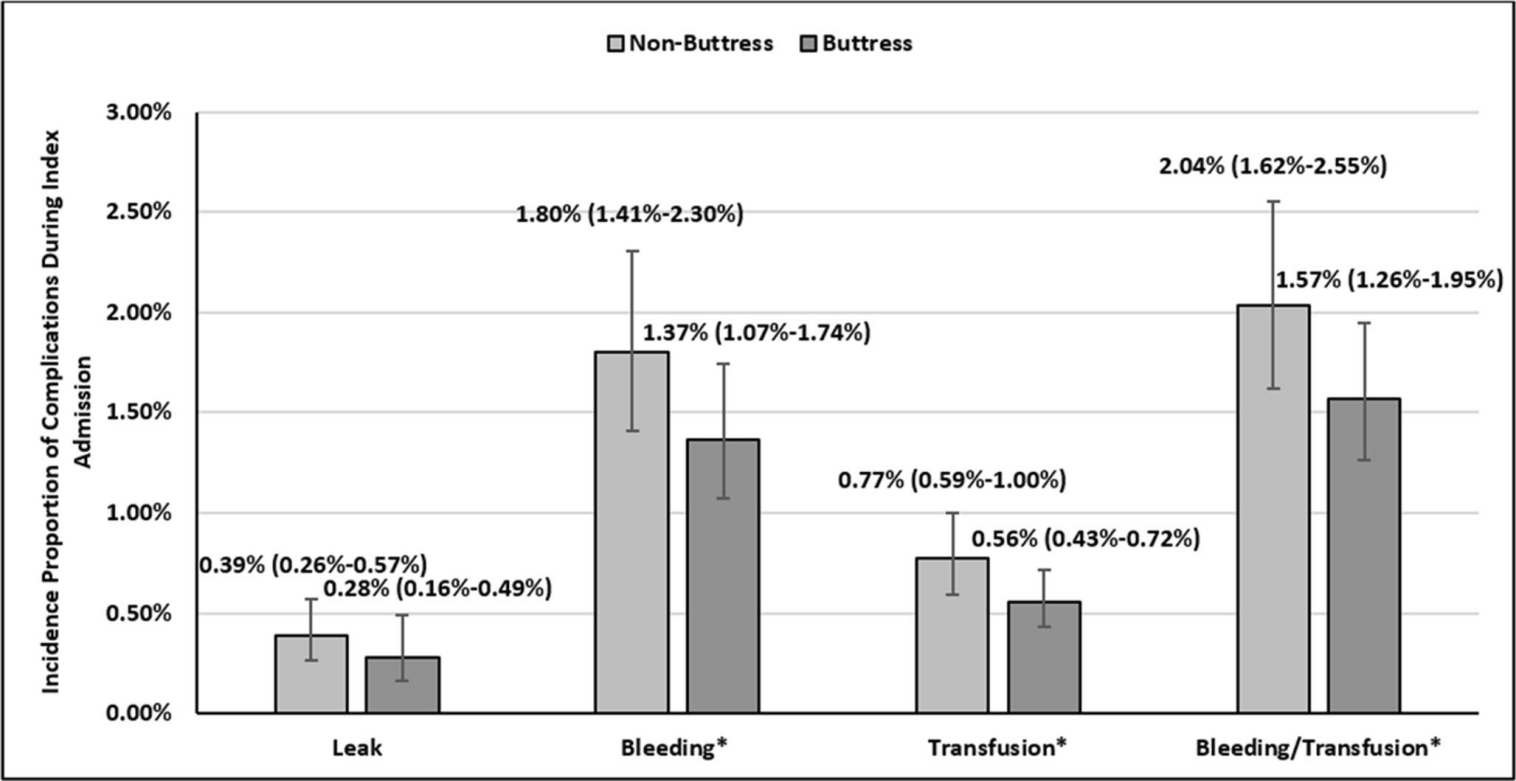
Surgical outcomes at index hospitalization among sleeve gastrectomy patients with vs. without buttress use. Mean incidence proportion (confidence limits); *p value < 0.05

After accounting for hospital-level clustering in the GEE models, staple line buttressing was associated with a lower rate of bleeding (1.37% vs 1.80%; *p* = 0.015), transfusion (0.56% vs 0.77%; *p* = 0.050), bleeding/transfusion (1.57% vs 2.04%; *p* = 0.019), and a similar rate of in-hospital leaks (0.28% vs 0.39%; *p* = 0.160), as compared with no buttressing during the index hospitalization (Fig. 3).

### Hospital Utilization and Costs

Before PSM, the average index hospitalization length of stay (1.75 vs 1.70 days; SMD = 1.7) and operation room time (2.85 vs 3.09 hours; SMD = 8.7) were similar between the buttress and non-buttress cohorts (Table 3).

**Fig. 4:**
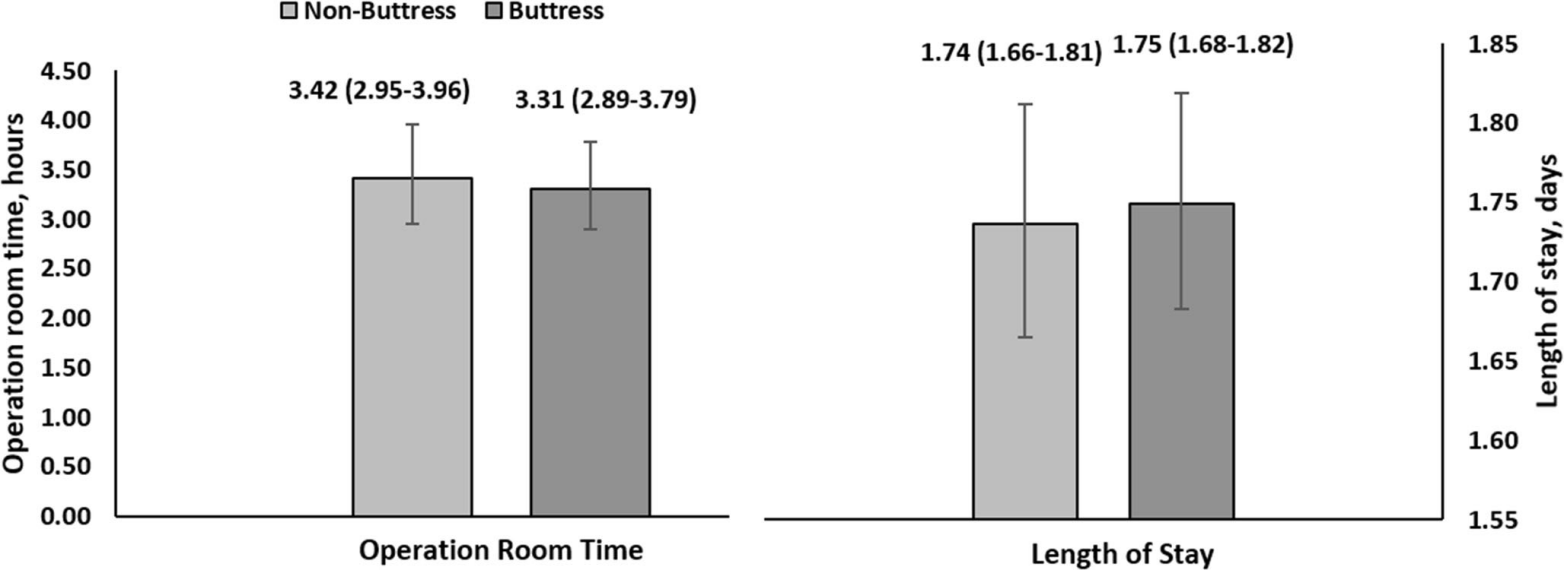
Index hospitalization utilization among sleeve gastrectomy patients with vs. without buttress use*. Mean time (confidence limits); *p value > 0.05

The index hospitalization LOS (1.75 vs 1.74 days; *p* = 0.718) and OR time (3.31 vs 3.42 h; *p* = 0.644) were similar between the buttress and non-buttress cohorts (Fig. 4).

Before PSM, the average total ($11,477 vs $10,712; SMD = 14.9), room and board ($1085 vs $993; SMD = 11.6), and supply costs ($4890 vs $4134; SMD = 22.3) were significantly higher, while operation room costs ($3752 vs $4040; SMD = 12.5) were lower in the buttress cohort (Table 3).

**Fig. 5:**
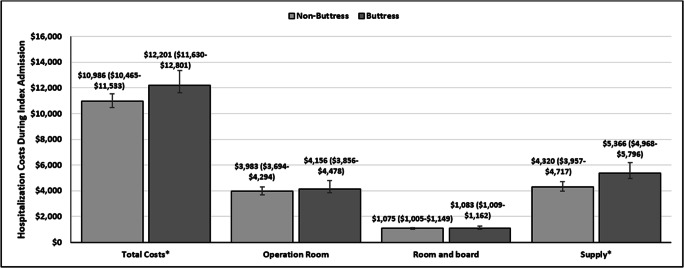
Hospitalization costs during index hospitalization among patients with and without buttress use. Mean costs (confidence limits); *p value < 0.05; adjusted to 2017 United States Dollars

Total health care costs during index hospitalization were higher by $1215 among patients in the buttress cohort ($12,201 vs 10,986; *p* < 0.001). That difference was primarily driven by medical and surgical supply costs, which were higher by $1046 in the buttress cohort ($5366 vs $4320; *p* < 0.001). OR ($4156 vs $3983; *p* = 0.132) and room and board costs ($1083 vs $1075; *p* = 0.793) were similar between the buttress and non-buttress cohorts (Fig. 5).

## Discussion

The study is one of the first real-world studies using a large nationwide US healthcare database to compare clinical and economic outcomes associated with buttress use among LSG patients.

After balancing potential confounders using PSM and accounting for hospital-level clustering in the GEE models, individual bleeding, transfusion, and composite bleeding/transfusion rates were lower by 0.43, 0.21, and 0.47 percentage points, respectively, among the buttress cohort patients compared with the non-buttress cohort, while total hospitalization costs were higher by $1215, driven primarily by supply costs.

Evidence from RCTs and prospective studies evaluating effectiveness of buttress materials have reported that buttressing staple lines reduces bleeding events [6, 7, 9, 12]. The study by Zafar et al. using Metabolic and Bariatric Surgery Accreditation and Quality Improvement Program dataset from 2015 evaluated postoperative bleeding among LSG buttress patients in the United States. The rate of postoperative bleeding was 0.80% for patients without suture over-sewing or buttressing and 0.57% for those with buttress use. After multivariable analyses, the buttress cohort had a 30% lower rate of bleeding (odds ratio = 0.70) [10]. In the current study, similar trends were found; a 0.23 percentage-point reduction in postoperative bleeding was observed among buttress cohort patients compared with those in the non-buttress cohort. A large multi-center study also reported post-operative bleeding rates of ≤ 1.7% among LSG patients, which are consistent with the current study [19].

There is inconclusive evidence available regarding reduction of leak rates with buttressing staple line among LSG patients. Results of RCTs available to-date could not make such inference owing to inadequate power [7]. Findings from a meta-analysis also remained inconclusive on the outcome among LSG patients [11]. The results of our study did not observe a statistically significant reduction in the rate of in-hospital leaks (0.28% vs 0.39%; *p* = 0.160) among buttress cohort patients compared with non-buttress cohort. Further research into materials that can improve leak rates could be highly beneficial in preventing the possibility of fatal complications such as septic shock ensuing from the complication [20].

Findings from an RCT showed a reduction in surgical time among patients with buttress use [12]. Though statistically insignificant, the buttress cohort was associated with lower OR time (3.31 vs 3.42 hours). The improvement in surgical time may be the result of lower rates of bleeding complications among patients in the buttress cohort [12].

A study conducted in Europe using absorbable buttress materials reported lower overall costs during the initial hospital stay in the buttress cohort [21]. Our study shows higher total costs among buttress cohort patients ($12,201 vs $10,986; *p* < 0.001). However, costs within sub-populations of absorbable and non-absorbable buttress materials were not evaluated in this study. In addition, variations in the costs of procedures and other health care expenses from study conducted in Europe may also have contributed to our conflicting findings. Further research of the cost-beneficial effects of absorbable buttress materials and other buttress materials is warranted.

In addition to the costs of buttress material reflected in medical and surgical supply costs ($5366 vs $4320; *p* < 0.001), higher total costs among buttress cohort patients in this study may also be related to unmeasured confounders not accounted for in the study. Providers with more buttress use may also have had a higher usage of other supplies along with buttressing, which may be responsible for the increase in medical and surgical supply costs. Accounting for confounders such as stapler type may elucidate the change in medical and surgical supply costs. Powered staplers are cost-effective compared with manual staplers, which incurred nearly $600 of additional costs, and their use can help improve cost savings from a policy perspective [22].

### Limitations

This study was subject to limitations. Errors or missing information in the database with respect to study variables and ICD-9/10-CM codes can lead to measurement error. The reasons for buttress use and the extent to which buttressing was used by each provider were not capturable in the dataset, and this could introduce selection bias. Residual confounding could exist because of factors such as cartridge and staple height selection [23]. Identification of leak outcomes was limited due to the lack of availability of specific ICD-9/10-CM codes for leak events. Codes used for identifying leak include peritoneal abscess, fistula of intestine (excluding rectum and anus), and other digestive system complications. Specific breakdowns of time in OR and skin-to-skin operation room times were not capturable in the dataset, and OR times should therefore be interpreted accordingly. For a given patient, the PHD longitudinally tracks only hospital encounters occurring within the same hospital and does not include complete information on follow-up visits, and subsequent medical visits of patients to other hospitals are not possible to track. Thus, the majority of information on patient characteristics was ascertained during the index hospitalization, and study outcomes were limited to those within the index hospitalization. In addition, this focus on index hospitalization also precluded capture of outcomes after discharge, including complications such as post-index leaks or bleeding, post-operative mortality, readmissions and reoperations, and costs. Likewise, the thromboprophylaxis practice and anticoagulation history of patients and their influence on surgical outcomes were out of the scope of the study. Further, the dataset did not support capture of certain procedural specifics, such as the use of double vs single-sided buttressing.

Moreover, no statistically significant difference was observed for in-hospital leaks between the buttress and non-buttress cohorts. However, PSM-adjusted results revealed that the buttress cohort had a higher rate of in-hospital leaks (0.42% vs 0.25%, SMD = 2.8) before applying the GEE model, but a lower rate after (0.28% vs 0.39%), which may indicate a correlation between in-hospital leak outcomes and providers.

As this is a non-randomized retrospective, observational study, results require caution in interpretation, only association rather than causation can be inferred. Finally, considering that the study used data from United States, our results may not be generalizable to populations outside the United States.

## Conclusion

Staple line buttressing was associated with an improvement in complication rates for bleeding and transfusion. Total costs and supply costs were higher in the buttress cohort, necessitating further research into cost-effective buttressing materials.

## Electronic supplementary material

ESM 1 (DOCX 26 kb)
