# Supplementary material for: Surgical Outcomes, Health Care Utilization, and Costs Associated with Staple Line Buttressing Among Primary Sleeve Gastrectomy Patients
Source: Obes Surg. 2020 Sep 10;30(12):4935–44. doi: 10.1007/s11695-020-04917-2 (PMC7719115; doi:10.1007/s11695-020-04917-2)
Supplement: Supplementary file 1 — (DOCX 26 kb) [file 11695_2020_4917_MOESM1_ESM.docx]

**Supplemental Table 1. Patient, Hospital, and Clinical Characteristics After Propensity Score Matching**

| **Baseline Variables** | **Non-Buttress Cohort** | | **Buttress Cohort** | | **SMD** |
| --- | --- | --- | --- | --- | --- |
|  | **(N= 24,049)** | | **(N= 24,049)** | |  |
|  | **N/Mean** | **%/SD** | **N/Mean** | **%/SD** |  |
| **Demographic Characteristics** | | | | | |
| **Age** | 44.4 | 11.9 | 44.5 | 12.0 | 1.5 |
| **18-34** | 5,351 | 22.3% | 5,376 | 22.4% | 0.2 |
| **35-54** | 13,605 | 56.6% | 13,292 | 55.3% | 2.6 |
| **55-64** | 3,762 | 15.6% | 3,965 | 16.5% | 2.3 |
| **65+** | 1,331 | 5.5% | 1,416 | 5.9% | 1.5 |
| **Race** | | | | | |
| **African American** | 4,405 | 18.3% | 4,732 | 19.7% | 3.5 |
| **Caucasian** | 16,501 | 68.6% | 15,966 | 66.4% | 4.8 |
| **Other race** | 2,795 | 11.6% | 3,188 | 13.3% | 5.0 |
| **Unknown race** | 348 | 1.4% | 163 | 0.7% | 7.5 |
| **Sex** | | | | | |
| **Male** | 5,162 | 21.5% | 5,281 | 22.0% | 1.2 |
| **Female** | 18,887 | 78.5% | 18,768 | 78.0% | 1.2 |
| **Marital Status** | | | | | |
| **Married** | 12,642 | 52.6% | 12,340 | 51.3% | 2.5 |
| **Single** | 9,615 | 40.0% | 9,922 | 41.3% | 2.6 |
| **Other** | 1,792 | 7.5% | 1,787 | 7.4% | 0.1 |
| **US Geographic Region** | | | | | |
| **Northeast** | 5,163 | 21.5% | 4,782 | 19.9% | 3.9 |
| **Midwest** | 4,123 | 17.1% | 4,687 | 19.5% | 6.1 |
| **South** | 12,088 | 50.3% | 11,539 | 48.0% | 4.6 |
| **West** | 2,675 | 11.1% | 3,041 | 12.6% | 4.7 |
| **Payer Type** | | | | | |
| **Managed care and commercial** | 15,755 | 65.5% | 15,447 | 64.2% | 2.7 |
| **Government (Medicare, Medicaid) and other government payers** | 6,523 | 27.1% | 6,793 | 28.2% | 2.5 |
| **Other (i.e., self-pay, workers’ compensation, direct employer contract, other)** | 1,749 | 7.3% | 1,803 | 7.5% | 0.9 |
| **Indigent and charity** | 22 | 0.1% | 6 | 0.0% | 2.8 |
| **Patient Clinical Characteristics** | | | | | |
| **Body Mass Index (BMI)** | | | | | |
| **<40** | 4,479 | 18.6% | 4,410 | 18.3% | 0.7 |
| **[40-45)** | 7,619 | 31.7% | 7,528 | 31.3% | 0.8 |
| **[45-50)** | 5,159 | 21.5% | 5,135 | 21.4% | 0.2 |
| **[50-60)** | 4,585 | 19.1% | 4,681 | 19.5% | 1.0 |
| **[60-70)** | 1,180 | 4.9% | 1,230 | 5.1% | 1.0 |
| **≥70** | 350 | 1.5% | 361 | 1.5% | 0.4 |
| **Missing BMI Information** | 677 | 2.8% | 704 | 2.9% | 0.7 |
| **Year of Surgery** | | | | | |
| **2012** | 1,925 | 8.0% | 1,995 | 8.3% | 1.1 |
| **2013** | 3,451 | 14.3% | 3,578 | 14.9% | 1.5 |
| **2014** | 4,691 | 19.5% | 4,684 | 19.5% | 0.1 |
| **2015** | 5,353 | 22.3% | 5,294 | 22.0% | 0.6 |
| **2016** | 5,349 | 22.2% | 5,179 | 21.5% | 1.7 |
| **2017** | 3,280 | 13.6% | 3,319 | 13.8% | 0.5 |
| **Deyo-Charlson comorbidity index score** | 0.65 | 0.97 | 0.70 | 0.99 | 4.8 |
| **0 (Reference)** | 13,291 | 55.3% | 12,727 | 52.9% | 4.7 |
| **1** | 7,747 | 32.2% | 7,937 | 33.0% | 1.7 |
| **2-3** | 2,543 | 10.6% | 2,864 | 11.9% | 4.2 |
| **4+** | 468 | 1.9% | 521 | 2.2% | 1.6 |
| **Hospital Characteristics** | | | | | |
| **Procedure volume (number of procedures/year)** | 782.9 | 436.1 | 769.5 | 395.5 | 3.2 |
| **Hospital Type** | | | | | |
| **Teaching** | 10,920 | 45.4% | 10,820 | 45.0% | 0.8 |
| **Non-teaching** | 13,129 | 54.6% | 13,229 | 55.0% | 0.8 |
| **Hospital Location** | | | | | |
| **Urban** | 22,485 | 93.5% | 22,113 | 91.9% | 6.0 |
| **Rural** | 1,564 | 6.5% | 1,936 | 8.1% | 6.0 |
| **Hospital Size** | | | | | |
| **<200 beds** | 4,797 | 19.9% | 4,961 | 20.6% | 1.7 |
| **200-500 beds** | 12,804 | 53.2% | 12,717 | 52.9% | 0.7 |
| **≥500 beds** | 6,448 | 26.8% | 6,371 | 26.5% | 0.7 |
| SD: standard deviation; SMD: standardized mean difference | | | | | |
